# Non-destructive assessment of apple internal quality using rotational hyperspectral imaging

**DOI:** 10.3389/fpls.2024.1432120

**Published:** 2024-11-06

**Authors:** Xiaojiang Wang, Junying Han, Chengzhong Liu, Tong Feng

**Affiliations:** ^1^ College of Information Science and Technology, Gansu Agricultural University, Lanzhou, China; ^2^ Research Institute of Pomology of Jingning Country, Pingliang, China

**Keywords:** hyperspectral imaging, apple, physicochemical property, machine learning, rotational acquisition method

## Abstract

This work aims to predict the starch, vitamin C, soluble solids, and titratable acid contents of apple fruits using hyperspectral imaging combined with machine learning approaches. First, a hyperspectral camera by rotating samples was used to obtain hyperspectral images of the apple fruit surface in the spectral range of 380~1018 nm, and its region of interest (ROI) was extracted; then, the optimal preprocessing method was preferred through experimental comparisons; on this basis, genetic algorithms (GA), successive projection algorithms (SPA), and competitive adaptive reweighting adoption algorithms (CARS) were used to extract feature variables; subsequently, multiple machine learning models (support vector regression SVR, principal component regression PCR, partial least squares regression PLSR, and multiple linear regression MLR) were used to model the inversion between hyperspectral images and internal nutrient quality physicochemical indexes of fruits, respectively. Through the comparative analysis of all the model prediction results, it was found that among them, for starch, vitamin C, soluble solids, and titratable acid content, 2^nd^ Der-CARS-MLR were the optimal prediction models with superior performance (the prediction coefficients of determination R_p_^2^ exceeded 90% in all of them). In addition, potential relationships among four nutritional qualities were explored based on t-values and p-values, and a significant conclusion was drew that starch and vitamin C was highly correlated.

## 1 Introduction

As one of the major fruits in the stable annual supply of global fruit markets, apples are highly valued for their market and nutritional qualities. Global demand for fresh apples and their processed products continues to grow, with consumers paying increasing attention to the internal quality of apples (Ma et al., 2018). With the innovation of fruit germplasm resources and the breeding of new special purposed apple varieties, the external quality of the fruit has been significantly improved, yet how to easily, quickly and accurately identify its internal quality has attracted great attentions from breeding and horticultural experts worldwide.

Currently, scientific researchers mainly use physical, chemical, molecular and biotechnological methods to systematically test and calibrate the internal nutritional quality and physicochemical indexes of apple fruits in the laboratory. Although this method is highly accurate, the fruit samples have to be destructively processed, which is expensive and time-consuming, and the process of processing and operation is both complicated and professional, which is not only not applicable to ordinary consumers but also not easy to be realized by general scientific researchers and horticultural workers. In addition, the use of chemical reagents may cause environmental pollution. Given this, there is an urgent need to develop an efficient and non-destructive assay to assess the internal nutritional qualities of apple fruits, which is of great significance in realizing the grading and marketing of apples, branding and industrial development (Mo et al., 2017).

HSI (Hyperspectral imaging) technology, as an emerging non-destructive testing method, has gained wide recognition in the field of fruit and vegetable quality assessment. This technique combines the strengths of spectral analysis and machine vision, and is able to synchronize the acquisition of spectral data and spatial distribution information of fruits and vegetables (Li et al., 2023). With HSI, the average spectral information of specific regions of fruits and vegetables can be accurately acquired, thus improving the robustness of the detection results (Liu et al., 2022). Recently, this technique has been applied in the internal qualities assessment of apples, e.g., Tian et al. combined HSI with an output correlation-based deep learning algorithm-stacked weighted adaptive encoder (SWAE) to detect the soluble solids content of Fuji apples (Tian et al., 2022). Ma et al. utilized HSI to detect the total sugar content (Ma et al., 2022), and Wang et al. predicted apple hardness using HSI (Wang et al., 2012). However, few studies have been reported on the HSI detection of vitamin C, titratable acid and starch content in apple fruits. Moreover, it is well known that nutrients in apples must be interrelated, and quality measurements of a single or a few indicators are insufficient to accurately reflect the overall quality (Shao et al., 2022). Apparently, the integrated consideration of multiple indicators more comprehensively reflects the quality of apple fruits, enabling a deeper and more systematic exploration of the apples’ potential value. In the existing research reports on fruit and vegetable quality, two of the internal nutritional qualities (hardness and soluble solids) are generally considered at most, e.g., Peng et al. utilized hyperspectral scattering technology combined with multivariate linear regression to predict the hardness and soluble solids content of apple fruits (Peng and Lu, 2008), Xie et al. utilized HSI technology combined with a new band extraction method to predict the color and hardness of banana (Xie et al., 2018) and Gao et al. predicted the hardness and soluble solids content of begonia fruit using HSI (Gao et al., 2022). Given this, this study used HSI combined with machine learning approaches to construct an inverse model of four major nutritional qualities within apples, accurately predict their contents and explore the relevant potential relationships among the four major nutritional qualities.

More than spectral data acquisition from a single angle is required to pursue comprehensiveness and accuracy in fruit quality inspection. Therefore, researchers in the field of HSI have advanced the development of imaging techniques capable of capturing images of samples in all directions in recent years. For example, Sun et al. achieved all-round (360°) imaging of peaches to assess their degree of decay by configuring a HSI system with a rotating platform (Sun et al., 2018). Similarly, the Ma et al. research team innovatively designed a rotating carrier platform-based HSI system applied to kiwifruit image acquisition as a means to assess its pH and soluble solids distribution (Ma et al., 2021). In addition, Shang et al. researched and developed a rotary clamping device with an integrated hyperspectral camera for detecting defects on navel orange surfaces (Shang et al., 2023). However, due to the variability of fruit size and morphology, it is not easy to maintain a constant speed during fruit rotation. Oscillations or wobbles may occur during fruit rotation, leading to data overlap or omission of spectral scan lines in specific regions (Pham and Liou, 2020). Given this, this study used a combination of fixed viewing angle and manual rotation to acquire hyperspectral images and, to reduce the negative effects of fruit shape, illumination conditions, and data overlap, did not use the entire fruit as the focus area of the study, but rather the center region of the apple fruit was used as the ROI.

As is well known, due to the shape and surface characteristics of apple fruits, the coloring of the apple surface is not completely uniform. This is primarily because the apple surface cannot receive light evenly, and there will always be a backlit side. This leads to variations in the nutritional content across the apple surface, with particularly large differences between the light-facing and backlit sides. Collecting spectral data from a single angle makes it difficult to accurately capture the true distribution of internal nutrients. To objectively and accurately model and assess the nutritional content of the entire fruit, we adopted a rotational acquisition method. By stably rotating the fruit, spectral information from all four sides was collected, providing a more comprehensive reflection of the entire apple’s true condition. Stable rotation is crucial for linear-array scanning imaging systems, as it improves image quality and reduces imaging errors caused by irregular shapes or unstable movements. This method aligns with the pursuit of comprehensiveness and precision in the field of hyperspectral imaging, ensuring the reliability of the study results.

In this study, a HSI method based on rotated samples is proposed, with the specific objectives of (1) utilizing HSI technology combined with a manual rotation method to obtain 360° spectral information of the whole apple surface; (2) comparing and selecting the optimal fusion of different preprocessing techniques, feature extraction methods, and machine learning methods to establish the optimal prediction model for inversion of four internal qualities of apple fruits; (3) based on the CARS-MLR model, the t-values and p-values were used to identify the characteristic bands related to starch, vitamin C, soluble solids and titratable acids in apple fruits, and the potential relationship between the four internal nutrient qualities was further explored.

## 2 Materials and methods

### 2.1 Sample preparation

The experimental samples were obtained from the orchard of Fruit Tree Research Institute, Jingning County, Gansu Province (35°28’N, 104°44’E, average altitude 1600 m). A total of five apple varieties, Chengji No. 1, Ruixue, Ruiyang, Yanfu No. 3, and Jingning No. 1, which are the main cultivars in Gansu Province, were selected and harvested at the beginning of October 2023 during the ripening period of the samples. Twelve fruit of each variety were selected from 12 different fruit trees listed and labeled, and the fruit samples with similar size, uniform coloring, no insect damage, and no mechanical damage were selected as much as possible. Immediately after harvesting, the fruit were protected with plastic foam, boxed, and transported back to the College of Information Science and Technology, Gansu Agricultural University laboratory. Numbered one by one, A_1∼A_12 represented Chengji No.1; B_1∼B_12 represented Ruixue; C_1∼C_12 represented Ruiyang; D_1∼D_12 represented Yanfu No.3; and E_1∼E_12 represented Jingning No.1.

### 2.2 Hyperspectral imaging acquisition and spectral extraction

#### 2.2.1 Hyperspectral imaging system

**Figure 1:**
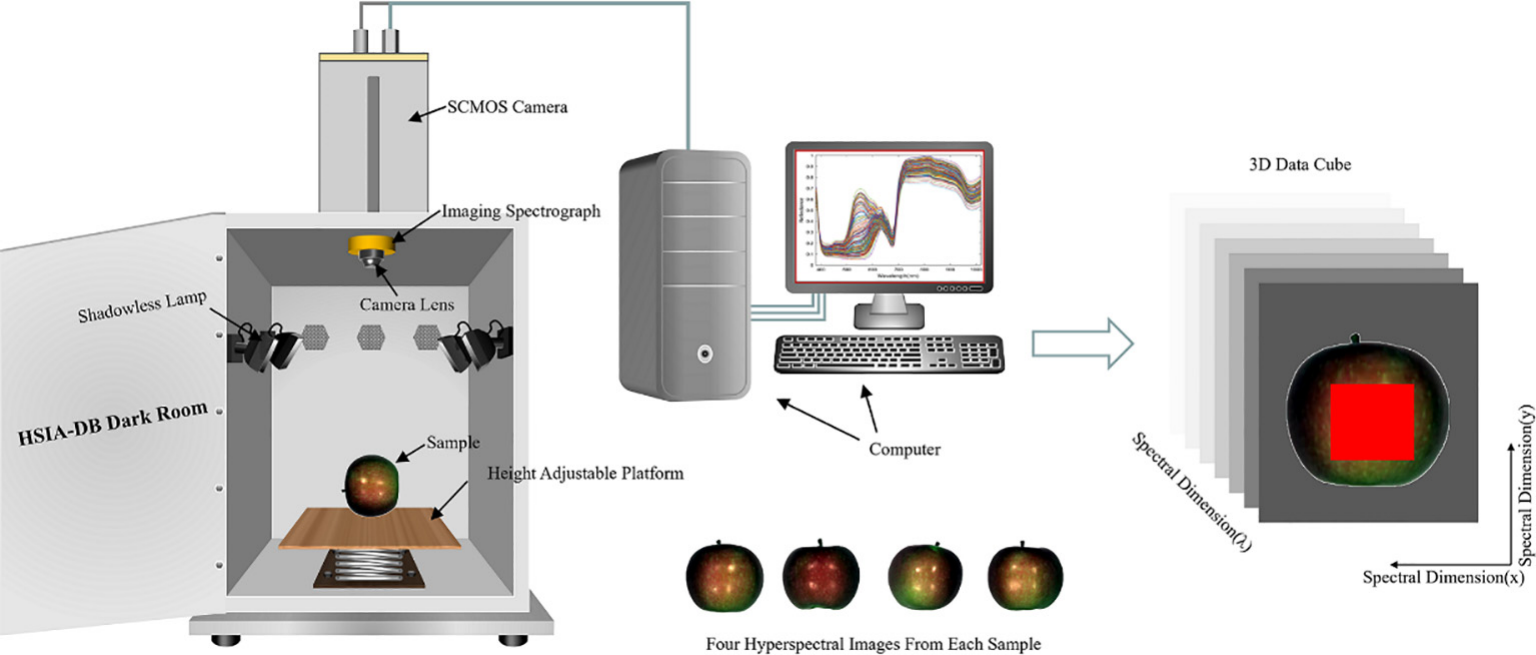
The hyperspectral imaging system and 3D data cube.

This experiment collected the reflectance data of 320 spectral bands in the 380-1018 nm range using a GaiaField portable hyperspectral system (Sichuan Dualix Spectral Imaging Technology Co., Ltd). The system consists of a GaiaField-V10E hyperspectral imager, an imaging spectrometer with a resolution of 2.8 nm, a 2048-pixel × 2048-pixel HSIA-GL16 imaging lens, four shadowless light sources (adjusted at an angle of about 30° to illuminate the field of view of the camera), and a height adjustable platform and data acquisition software (SpecView). The effective slit length is 14.2 mm, the slit width is 30 μm, the detector is SCMOS, and the numerical aperture is F/2.8. The HSI system operates on push-broom scanning, integrating a linear array detector with an imaging spectrometer. The motorized platform is engineered to facilitate scanning motion, causing the imaging spectrometer’s entrance slit to traverse across the focal plane of the imaging lens, thereby enabling the capture of hyperspectral data. The detector captures real-time spectral data corresponding to the linear target, compiled into a comprehensive data cube, as depicted in **Figure 1**.

#### 2.2.2 Acquisition of hyperspectral image information

Accurate adjustment of acquisition parameters is crucial to capture high-quality, undistorted hyperspectral imagery. In this study, the distance between the SCMOS lens and the carrier stage was set to 40 cm, and the camera’s exposure time, gain mode, and actual frame rate were set to 49 ms, 2, and 18 mm/s, respectively. The samples must be placed in an HSIA-DB dark box during the data acquisition to avoid the interference of stray rays. Four spectral acquisitions were performed for each apple sample to analyze and process the full range of data. One apple sample was placed on the carrier stage to collect spectra, keeping the petiole-calyx axis parallel to the carrier stage so that the center was aligned with the camera. For the second, third, and fourth spectra acquisition, the apple sample was rotated 90 degrees around its petiole-calyx axis in the same direction each time until all four images were acquired. Hyperspectral images for 60 apple samples were systematically acquired using a consistent methodology, resulting in a dataset comprising 240 hyperspectral images. After imaging, the apple specimens were dispatched to Suzhou Dream Rhinoceros Biomedical Technology Co. Ltd. for detailed analysis of their VC, TA, SSC, and starch content. **Table 1** lists the maximum, minimum, average, and standard deviation of each parameter.

**Table 1:** Presentation of physical and chemical data of samples.

Parameters	Maximum	Minimum	Average	Standard deviation
Starch(%)	10.88	4.25	7.43	2.69
VC(‰)	2.30	1.02	1.90	0.43
SSC(%)	16.00	11.40	13.27	1.60
TA(‰)	5.14	2.45	3.41	0.99

To eliminate the effects of noise due to differences in the shape of the apples, the uneven distribution of the light source intensity under each wavelength band, and the presence of dark current in the camera, the acquired raw hyperspectral image (R_origin_) needs to be corrected for black and white. The image is first acquired on a standard white correction plate made of PTFE material to obtain an all-white calibration image (R_white_), and then an all-black calibration image (R_dark_) is acquired. The corrected hyperspectral image (R) was obtained using Equation 1.


(1) $$\begin{array}{c} R=\frac{R_{origin}-R_{dark}}{R_{white}-R_{dark}} \end{array}$$

#### 2.2.3 Spectral extraction

For the calibrated hyperspectral images, 480 pixels × 480 pixels ROI hyperspectral data were extracted using ENVI 5.6 software. The abnormal information due to the light on a particular place of the apple samples was also eliminated in the extraction of ROI. Finally, the average of all the spectral information within the ROI was used as the corresponding reflectance spectral value.

To ensure the accuracy of the region of interest (ROI), we chose the Region Growing Algorithm to eliminate abnormal information. Initially, an area unaffected by abnormal information was selected as the seed point, and then the ROI was gradually constructed by expanding similar pixels. The reason why we chose the Region Growing Algorithm is that it can effectively handle irregular shaped ROIs and reduce abnormal interferences by lighting reflection, sample curvature, or other factors. The specific parameters in this study are as follows: Growth Size was set to 480 × 480 pixels, the Std Dev Multiplier was set to 2.2, and the number of iterations was set to 50.

### 2.3 Physicochemical properties measurement

#### 2.3.1 VC content

First, 10 ml of ascorbic acid standard solution was extracted with 30 ml of 2% oxalic acid solution, titrated with 2.6-dichlorophenol indophenol solution until the pink color lasted for 30 seconds as the endpoint, and the amount of dye was recorded to determine the titer. The sample was treated similarly: 10 ml of filtrate was taken for titration, and VC was calculated according to Equation 2.


(2) $$\begin{array}{c} VC\left(‰\right)=\left(v_{3}-v_{2}\right)\times d/m\times v_{t}/v_{s}\times 100 \end{array}$$

where v_2_ and v_3_ denote the volume of dye used to titrate the blank versus the sample, respectively, d denotes the degree of titration, m denotes the weight of the sample, v_t_ denotes the total volume of the extracted sample solution, and v_s_ denotes the volume of the sample used in the titration.

#### 2.3.2 TA content

Prepare homogenate by taking 250g of the edible portion of the specimen and mashing it with an equal amount of water. Take 50-100g of homogenate, wash with water into a 250mL volumetric flask, heat to 75-80°C, shake, calm, and then set the volume and filter. Take 50 or 100mL sample solution, add phenolphthalein indicator, and titrate with sodium hydroxide standard solution until slightly red color for 30 seconds without retreating. Calculate the TA according to Equation 3.


(3) $$\begin{array}{c} TA\left(‰\right)=c\times \left(v_{1}-v_{0}\right)\times k\times F/m\times 1000 \end{array}$$

where c denotes the concentration of sodium hydroxide solution, v1 denotes the volume consumed for the sample’s titration, v0 denotes the volume used in the blank titration, k denotes the acid conversion factor, and m denotes the mass of the specimen.

#### 2.3.3 SSC

Take the edible part of the sample chopped, mixed (frozen products must be thawed beforehand), weighing 250g, accurate to 0.1g, into the high-speed tissue masher mashing machine, with two layers of microscope paper or gauze extruded homogenized juice determination. These refractometer readings are the SSC.

#### 2.3.4 Starch content

Glucose standard series were prepared at the concentrations of 0, 0.2, 0.4, 0.6, 0.8, and 1.0 mg, using DNS reagent to develop the color, and the absorbance was measured at a specific wavelength; the standard curve was plotted, and the linear equation was derived. The absorbance was measured after the sample was treated similarly, and the sugar content was calculated by standard curve. Starch content was calculated according to Equation 4.


(4) $$\begin{array}{c} Starch\left(\%\right)=c\times v_{t}/v\times d/m\times 0.9 \end{array}$$

where c denotes the sugar content of the sample, vt denotes the total volume of the extract, v denotes the assay volume, d denotes the number of dilutions, m denotes the weight of the sample, and 0.9 denotes the starch hydrolysis correction factor.

### 2.4 Spectral data processing

Hyperspectral data are susceptible to disturbances such as spectral intensity differences, stray light, noise, and baseline drift (Xu et al., 2023). Therefore, several preprocessing methods were used in this study. Namely, Savitzky-Golay (SG) smoothing, moving average (MA) smoothing, normalization (NM), multiplicative scatter correction (MSC), baseline correction, standard normal variant transform (SNV), detrend (DT), first-order derivative (1^st^ Der) and second-order derivative (2^nd^ Der) (Mo et al., 2017; Tian et al., 2022). SG removes noise by fitting a polynomial within a given window to estimate the smoothed values of the data points. MA reduces noise and attenuates the volatility of the data by calculating the average of the neighboring data points in the spectral data series. NM scales the data into a specific range, usually [0, 1] or [-1, 1]. MSC aims to correct for spectral intensity variations due to light scattering effects, making the spectra more comparable and stable. The baseline aims to remove background noise and baseline drift from the spectral curves. SNV focuses on normalizing the spectral data at each wavelength point, i.e., centering and normalizing the data at each wavelength point, which helps to identify the differences between samples better. DT reduces the effect of external noise on the spectral curve by subtracting the trend line adapted to the noise component. 1^st^ Der is usually used to highlight the edges, peaks, and wavelength variations of the spectrum, while 2^nd^ Der is often used to locate the peaks and valleys in the spectra; in addition, they can help to eliminate baseline drift in the data and enhance the smaller-scale features in the spectral data.

### 2.5 Feature selection algorithms

Since the preprocessed data have many covariant wavelengths to the extent of information redundancy, it increases the computational volume and reduces the modeling efficiency. In this experiment, the Genetic Algorithm (GA), Sequential Projection Algorithm (SPA), and Competitive Adaptive Reweighted Sampling (CARS) algorithm are used to extract spectral data features in addition.

GA is a heuristic optimization method inspired by Darwin’s idea of evolution. GA evolves by modeling evolutionary processes such as natural selection, crossover, and mutation to generate feasible solutions. In GA, by genetically coding individuals in a population (representing candidate solutions in the solution space), operations such as selection, crossover, and mutation are used to generate the next generation of the population, and these individuals are gradually optimized to find the optimal solution. The core idea of GA lies in searching for optimal solutions from many solution spaces by modeling natural selection and genetic mechanisms (Lee et al., 2022). The specific parameters of the Genetic Algorithm are set as follows: the population size is set to 50, that is, 50 individuals are optimized in each generation; the maximum number of iterations is set to 100, and if the fitness of the best individual in the population has not been obviously improved for 20 consecutive iterations, the algorithm will be terminated ahead of schedule; the selection strategy adopts the roulette selection method; the probability of the crossover operation is 0.8 to ensure that 80% of the individuals in the population exchange genes to generate the next generation to keep the diversity of the population; the probability of the mutation operation is 0.01, which is used to introduce a small amount of random changes in each generation to prevent the algorithm from falling into a local optimum.

SPA is a forward iterative search method for solving covariance problems. The iterative process commences with the selection of a single wavelength, incrementally integrating a novel variable at each subsequent iteration. This continues until the cumulative variables reach the predetermined threshold, N. SPA aims to choose the wavelength with the least redundant spectral information. By iterating step by step, SPA can effectively select the wavelength with the best mutual information, thus improving the accuracy and stability of feature selection. By reducing the redundant information, the SPA algorithm can provide better spectral resolution performance and improve the feature selection (He et al., 2023).

The CARS approach integrates Monte Carlo sampling with PLS model regression coefficients, drawing inspiration from the concept of ‘survival of the fittest’ as postulated by Darwin. The algorithm employs an Adaptive Random Sampling (ARS) strategy to form a new subset, iteratively retaining points with higher weights based on the absolute values of PLS regression coefficients and discarding those with lower weights. Following this selection, a new PLS model is constructed and refined multiple times. The final feature wavelengths are chosen from the subset that minimizes the Root Mean Square Error of Cross-Validation (RMSECV). The CARS algorithm can efficiently select feature wavelengths with predictive solid ability through this iterative process, thus improving the accuracy and robustness of feature selection (Shao et al., 2021).

### 2.6 Regression models

Support Vector Regression (SVR) is a supervised learning technique derived from Support Vector Machines (SVM). It leverages kernel functions to transform vectors from a lower-dimensional space to a higher-dimensional space, where it constructs linear decision functions. This enables SVR to effectuate nonlinear decision-making in the original space (Balabin and Lomakina, 2011).

PCR involves transforming the original set of correlated variables into a new set of uncorrelated variables known as principal components (Sun, 1995). Subsequently, a regression model is constructed utilizing these principal components as predictors. PCR has become a valuable and powerful multivariate correction method for chemometric analysis, integrating independent and dependent variables (Araújo et al., 2001).

PLSR is a commonly used statistical modeling method for solving the problem of high correlation between independent variables in multiple linear regression. It reduces the effects of covariance among independent variables. It improves the stability and predictive power of the model by transforming the original independent variables into a new set of composite variables (principal components or latent variables) and then performing regression analysis on these principal components (Badaró et al., 2020).

MLR is a kind of relationship model that can build multiple independent variables and dependent variables, and MLR is applicable when the number of samples is more than the number of variables, so this study only builds this model for the wavelengths extracted by GA, SPA and CARS algorithms (Rajkumar et al., 2012).

### 2.7 Evaluation of model performance

**Figure 2:**
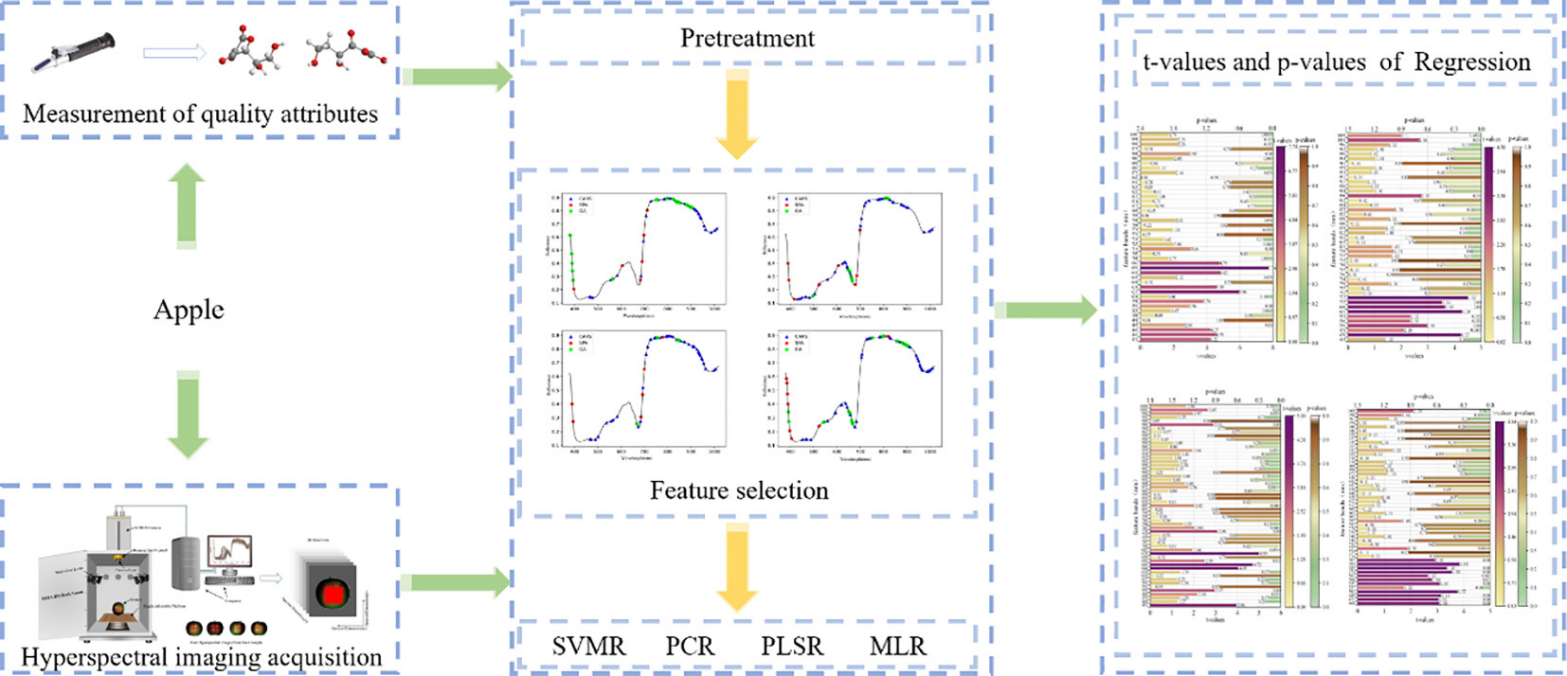
Experimental procedure.


The model performance was assessed by the following metrics: the coefficient of determination of the correction ( $R_{C}^{2}$ ) and prediction ( $R_{p}^{2}$ ) and the root mean square error of the correction (RMSEC) and prediction (RMSEP). In general, the higher the coefficient of determination and the smaller the root-mean-square difference, the better the performance of the model (Xiang et al., 2022). The derivation of the above parameters is shown in Equation 5 and Equation 6. The processing of the whole experiment is shown in **Figure 2**.

(5) $$\begin{array}{c} R^{2}=1-\frac{\sum_{i-1}^{n}{\left(y_{i}-{\hat{y}}_{i}\right)}^{2}}{\sum_{i-1}^{n}{\left(y_{i}-{\bar{y}}_{i}\right)}^{2}}\in \left[0,1\right] \end{array}$$

(6) $$\begin{array}{c} RMSE=\sqrt{\frac{1}{n}\sum_{i-1}^{n}{\left(y_{i}-{\hat{y}}_{i}\right)}^{2}} \end{array}$$

## 3 Results and analyses

### 3.1 Spectral profile

**Figure 3:**
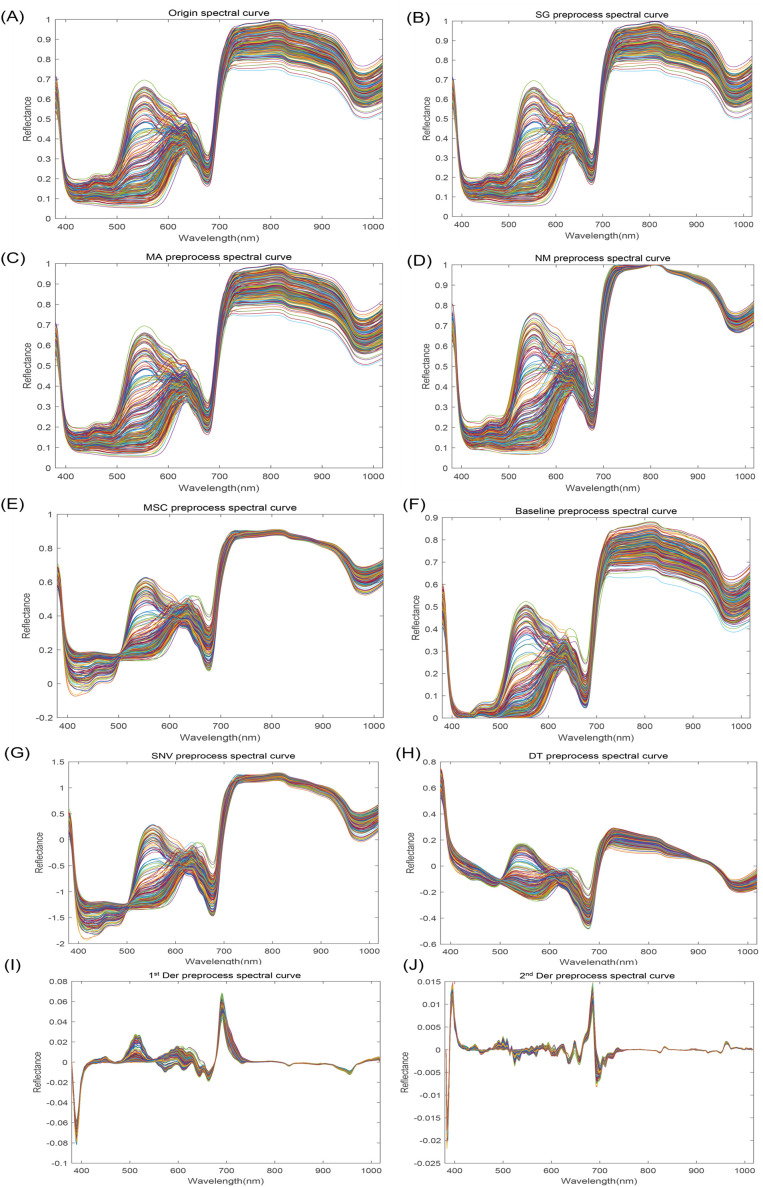
Apple spectral reflectance curves. **(A)** Origin spectral curve; **(B)** SG preprocess spectral curve; **(C)** MA preprocess spectral curve; **(D)** NM preprocess spectral curve; **(E)** MSC preprocess spectral curve; **(F)** Baseline preprocess spectral curve; **(G)** SNV preprocess spectral curve; **(H)** DT preprocess spectral curve; **(I)** 1^st^ Der preprocess spectral curve; **(J)** 2^nd^ Der preprocess spectral curve.

Spectral characterization of 320 bands within 380-1018nm was carried out to obtain all spectral curves of apple samples. **Figure 3A** illustrates that the average spectral curves for various apple cultivars exhibit similar trends. However, a pronounced discrepancy is observed in the spectral curves between 530 and 630 nm, suggesting notable variations in the constituent contents among apple varieties. Specifically, the observed spectral absorption peaks at approximately 500 nm and 680 nm can be primarily attributed to the characteristic absorption spectra of carotenoids and chlorophylls, respectively (Siedliska et al., 2014; Yu et al., 2014). As ripe apples contain more chlorophylls and the color information is reflected in the surface of apples, the spectral curves have more prominent absorption peaks at 680 nm (Song et al., 2024). The absorption peaks around 840 nm and 975 nm are related to the water and sugar inside the apple, which are O-H tertiary and secondary octave characteristic absorption peaks, respectively (Huang et al., 2013; Shao et al., 2020; Xue et al., 2013).

### 3.2 Selection of optimal preprocessing

Multiple preprocessing methods (**Figures 3B–J**) were compared to obtain the best one ultimately, and raw HSI data was preprocessed in section 2.4, based on which the starch content of apple samples was predicted using PLSR. As can be seen from **Table 2**, 2^nd^ Der is the optimal preprocessing method. Therefore, the 2^nd^ Der preprocessing method was used for further feature extraction to predict VC, TA, SSC, and starch. However, it is worth noting that since the direct derivation of HSI data may lead to the amplification of spectral noise, which may produce unpredictable results, the data were smoothed to minimize the effect of spectral noise before the derivation of the data for preprocessing.

**Table 2:** The prediction results of starch content are based on the original spectrum and different preprocessing methods in the PLSR model.

Methods	Calibration		Prediction	
	$R_{C}^{2}$	RMSEC	$R_{p}^{2}$	RMSEP
Origin data	0.8265	1.0972	0.8025	1.1768
SG	0.8661	0.9639	0.8460	1.0377
MA	0.8652	0.9672	0.8500	1.0236
NM	0.8839	0.8973	0.8590	0.9941
MSC	0.8742	0.9342	0.8476	1.0336
Baseline	0.8752	0.9306	0.8509	1.0201
SNV	0.8368	1.0641	0.8127	1.1438
DT	0.8766	0.9251	0.8577	0.9974
1^st^ Der	0.9030	0.8200	0.8743	0.9373
**2^nd^ Der**	**0.9378**	**0.6566**	**0.9034**	**0.8232**

Values in bold indicate the optimal model as well as his prediction results.

### 3.3 Extraction of spectral feature wavelength

**Figure 4:**
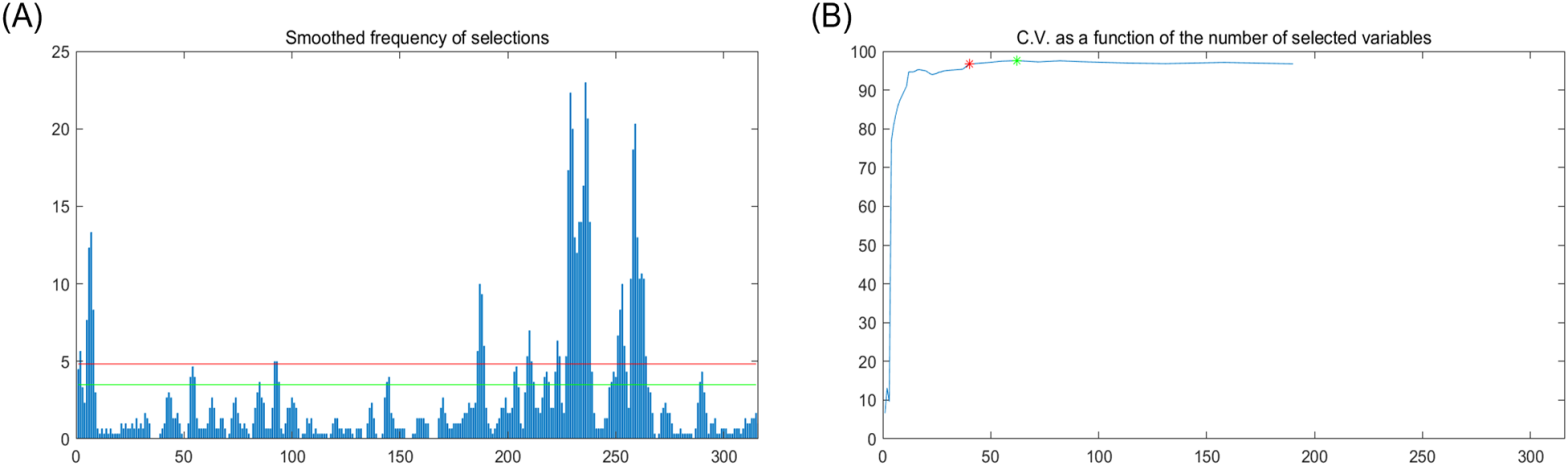
Feature wavelength selection graph based on GA algorithm. **(A)** Distribution of the feature variable, **(B)** Trend of the feature variable CV.

**Figure 4** shows the wavelength selection process of apple starch by the GA. **Figure 4A** indicates the number of times the feature wavelengths were selected after the code was run 100 times, and **Figure 4B** demonstrates the number of explained variances of the latent variables, where the green dots indicate the global maximum and the red dots indicate the best latent variables within the hypothesis testing limits. Finally, 20 feature wavelength variables were preferred, accounting for 6.25% of the total spectral number.

**Figure 5:**
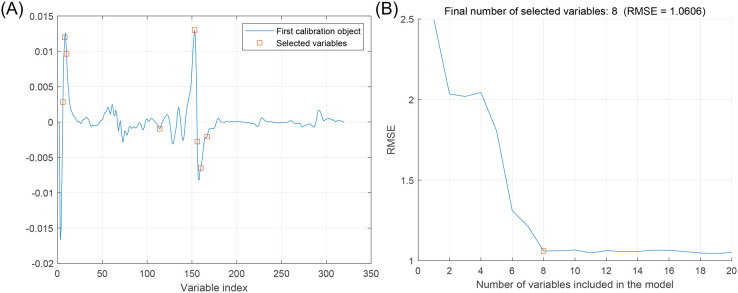
Feature wavelength selection graph based on SPA algorithm. **(A)** Distribution of feature variables, **(B)** Trends in RMSE of feature variables.

**Figure 5** shows the wavelength process of apple starch preferred by the SPA, specifying the number of variables N = 1 ~ 20. **Figure 5A** shows the distribution of selected wavelengths, and **Figure 5B** shows that the RMSE value decreases as the number of variables increases. The RMSE is the most minor overall when the number of variables is 8, which is 2.5% of the total spectra.

**Figure 6:**
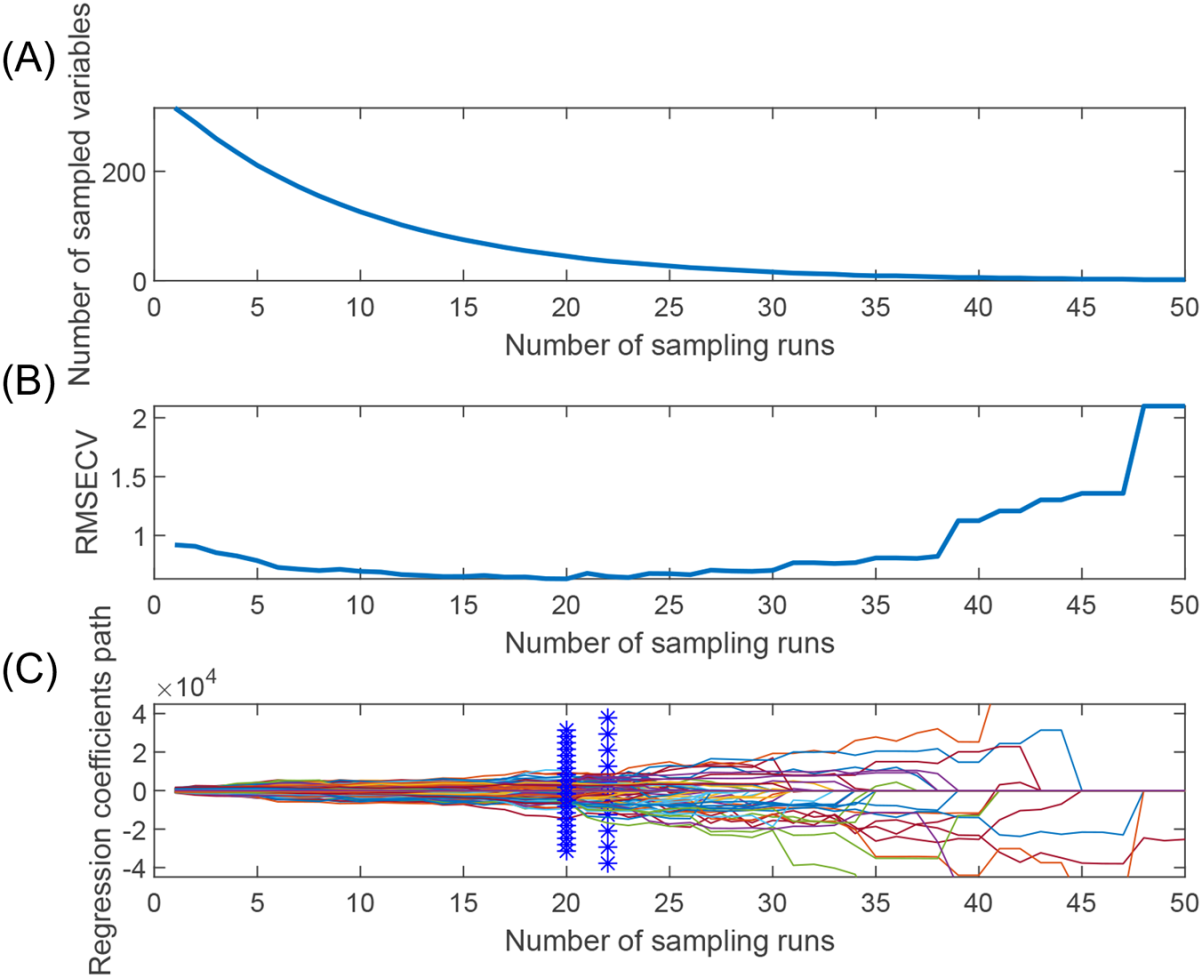
Feature wavelength selection graph based on CARS algorithm. **(A)** Trends in the number of feature variables, **(B)** Trends in the RMSEV values of feature variables, and **(C)** Trends in the path of regression coefficients.

**Figure 6** shows the process of optimizing the characteristic wavelength of apple starch using the CARS algorithm, in which the Monte Carlo sampling number was set to 50, and the characteristic wavelength was extracted using the 10-fold cross-validation method. **Figure 6A** shows two different stages of fast screening and fine screening in the process of wavelength preference by the CARS algorithm. The curve (number of selected wavelengths) decreases sharply in the initial fast screening stage. Subsequently, it enters the delicate screening stage, and the curve trend begins to smooth out. **Figure 6B** shows that the RMSECV values of the PLS model are gradually eliminated as the number of samples increases, leading to unimportant wavelengths. However, inevitably, some effective wavelengths are also removed, thus leading to larger RMSECV values (after the 19th sampling). The curve in **Figure 6C** shows the regression coefficients at different sampling times. Finally, 45 characteristic wavelength variables were preferred for apple starch analysis, which accounted for 14.06% of the total spectrum number.

In addition, the number of feature wavelengths and wavelength points obtained by the GA algorithm is inconsistent from run to run, and this instability adversely affects the model. In contrast, the wavelength points selected by the SPA algorithm are usually located near the peaks or troughs of the spectrum and are fewer in number. In addition, the SPA and CARS algorithms produced the same results for each run. From the above number of wavelengths after feature extraction for starch content, it can be seen that SPA facilitates the simplification and stabilization of the model for further modeling compared to the GA and CARS algorithms.

**Figure 7:**
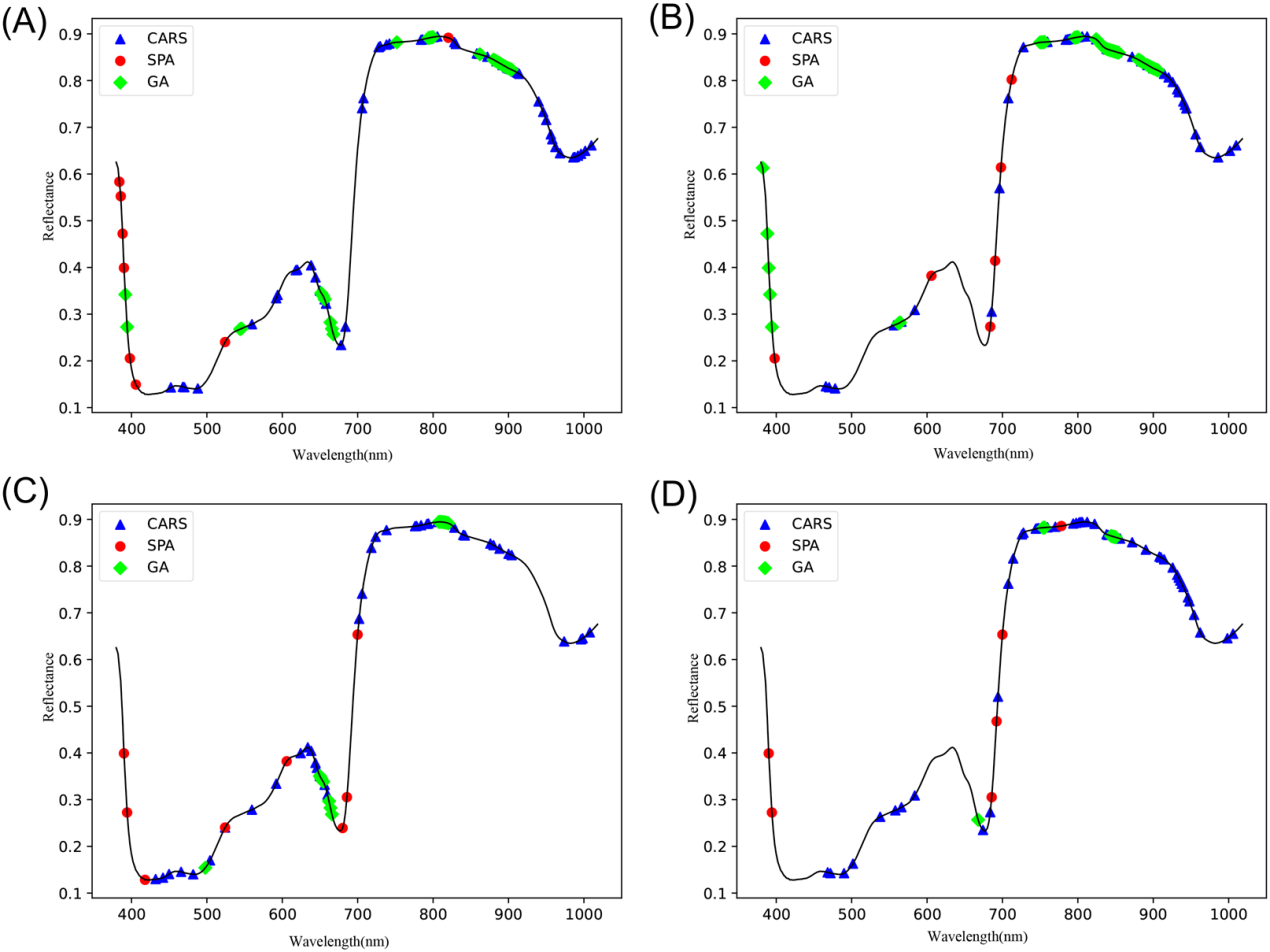
Distribution of feature wavelengths. **(A)** starch, **(B)** VC, **(C)** SSC, **(D)** TA.

The remaining physicochemical characteristics (VC, SSC, and TA) were also subjected to feature variable extraction using the exact parameters of GA, SPA, and CARS, and the feature wavelength distributions are shown in **Figure 7**.

### 3.4 Effect of feature selection on models

#### 3.4.1 Prediction of starch content

**Figure 8:**
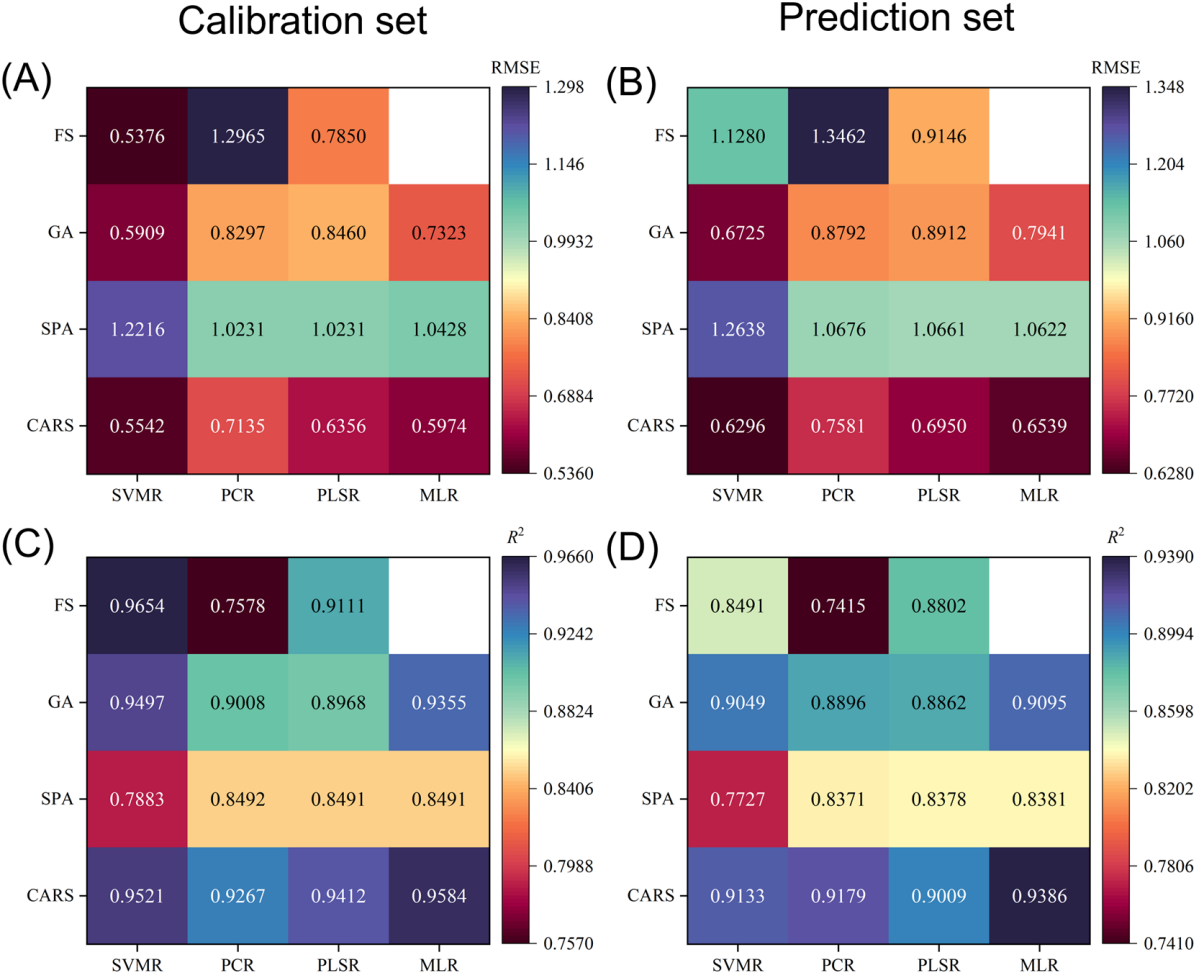
Prediction results of starch content for the calibration set **(A, C)** and prediction set **(B, D)** obtained by different calibration models using full spectral bands and spectral bands after feature extraction. Blanks represent MLR without the full spectral bands.

The prediction results of starch content are shown in **Figure 8**. The results from **Figures 8B, D** indicate the effect of using a feature extraction algorithm to extract feature wavelengths as input parameters for SVR, PCR, PLSR, and MLR models: CARS>GA>FS>SPA. In detail, the R^2^-value in the prediction set becomes more significant while the RMSE becomes concurrently smaller. Similarly, the same is true for **Figures 8A, C**. It is mentioned in Section 3.4 that SPA facilitates model simplification, stabilization, and further modeling relative to GA and CARS algorithms. It is worth noting that the SPA algorithm fails to improve the prediction performance and even reduces the prediction performance in some cases, although it reduces the information redundancy. This suggests that SPA may exclude information critical for accurate model prediction, thus affecting the performance of image validity prediction models. This shows that not all feature extraction algorithms positively impact the effectiveness of image prediction models. In addition, from **Figure 8D**, the optimal prediction model for apple starch content was 2^nd^ Der-CARS-MLR, with a prediction ensemble R^2^ of 0.9386 and an RMSE of 0.6530. Therefore, HSI of rotated samples in combination with 2^nd^ Der-CARS-MLR can be used as a non-destructive and efficient method for detecting the starch content of apple fruit.

#### 3.4.2 Prediction of VC, SSC, and TA

The results of the prediction of VC, SSC, and TA of apple fruit are shown in **Table 3**. As can be seen from **Table 3**, the optimal model used to predict VC: $R_{V}^{2}$ = 0.9088, RMSEV = 0.1245; the optimal model used to predict SSC, $R_{V}^{2}$ = 0.9248, RMSEV = 0.4213; and the optimal model used to predict TA: $R_{V}^{2}$ = 0.9683, RMSEV = 0.1678. It is worth noting that the optimal models used to predict VC, SSC, and TA were all one model and the same as those used to predict starch content, i.e., 2^nd^ Der-CARS-MLR. Therefore, the HSI of rotated samples combined with 2^nd^ Der-CARS-MLR can be used as a non-destructive and efficient way to detect VC, SSC, and TA in apple fruit.

**Table 3:** The prediction results of VC, SSC, and TA were obtained by different calibration models using full spectral bands and bands after feature extraction.

Parameters	Models	Extraction methods	No. of wavelength	Calibration		Prediction	
				$R_{C}^{2}$	RMSEC	$R_{p}^{2}$	RMSEP
VC	SVMR	FS	320	0.9331	0.1078	0.8772	0.1330
		GA	7	0.7768	0.2091	0.7619	0.2186
		SPA	6	0.6940	0.2399	0.6667	0.2496
		CARS	50	0.9185	0.1177	0.8962	0.1250
	PCR	FS	320	0.4561	0.3036	0.4308	0.3116
		GA	7	0.7758	0.1949	0.7627	0.2013
		SPA	6	0.7645	0.1997	0.7509	0.2062
		CARS	50	0.7437	0.2084	0.7311	0.2142
	PLSR	FS	320	0.8560	0.1562	0.8063	0.1822
		GA	7	0.7705	0.1972	0.7559	0.2046
		SPA	6	0.7645	0.1997	0.7549	0.2053
		CARS	50	0.9293	0.1094	0.8843	0.1210
	MLR	FS	/	/	/	/	/
		GA	7	0.7759	0.1982	0.7600	0.2021
		SPA	6	0.7645	0.2027	0.7504	0.2061
		**CARS**	**50**	**0.9406**	**0.1130**	**0.9088**	**0.1245**
SSC	SVMR	FS	320	0.9893	0.1909	0.9041	0.4203
		GA	14	0.8521	0.5971	0.8302	0.6359
		SPA	10	0.8549	0.5942	0.8336	0.6333
		CARS	44	0.9602	0.3133	0.9161	0.4212
	PCR	FS	320	0.6461	0.9126	0.6163	0.9540
		GA	14	0.7451	0.7744	0.7275	0.8026
		SPA	10	0.8148	0.6601	0.7937	0.6987
		CARS	44	0.8818	0.5212	0.8489	0.5988
	PLSR	FS	320	0.9233	0.4246	0.8658	0.5638
		GA	14	0.7912	0.7009	0.7695	0.7398
		SPA	10	0.8148	0.6601	0.7902	0.7064
		CARS	44	0.9279	0.4118	0.9079	0.4686
	MLR	FS	/	/	/	/	/
		GA	14	0.8010	0.7068	0.7660	0.7436
		SPA	10	0.8148	0.6758	0.7921	0.7008
		**CARS**	**44**	**0.9511**	**0.3760**	**0.9248**	**0.4213**
TA	SVMR	FS	320	0.9823	0.1312	0.9313	0.1948
		GA	29	0.9638	0.1801	0.9258	0.1989
		SPA	9	0.8938	0.3109	0.8820	0.3271
		CARS	55	0.9781	0.1400	0.9433	0.1736
	PCR	FS	320	0.8505	0.3638	0.8417	0.3758
		GA	29	0.9317	0.2458	0.9281	0.2533
		SPA	9	0.8705	0.3386	0.8585	0.3547
		CARS	55	0.9454	0.2198	0.9401	0.2317
	PLSR	FS	320	0.9604	0.1871	0.9412	0.2290
		GA	29	0.9111	0.2805	0.9027	0.2945
		SPA	9	0.8141	0.4057	0.8008	0.4217
		CARS	55	0.9489	0.2127	0.9422	0.2274
	MLR	FS	/	/	/	/	/
		GA	29	0.9573	0.2077	0.9437	0.2236
		SPA	9	0.8705	0.3459	0.8518	0.3552
		**CARS**	**55**	**0.9808**	**0.1486**	**0.9683**	**0.1678**

‘/’ indicates that MLR is not performed in the full-spectrum band. Values in bold indicate the optimal model as well as his prediction results.

#### 3.4.3 Model explanation

**Figure 9:**
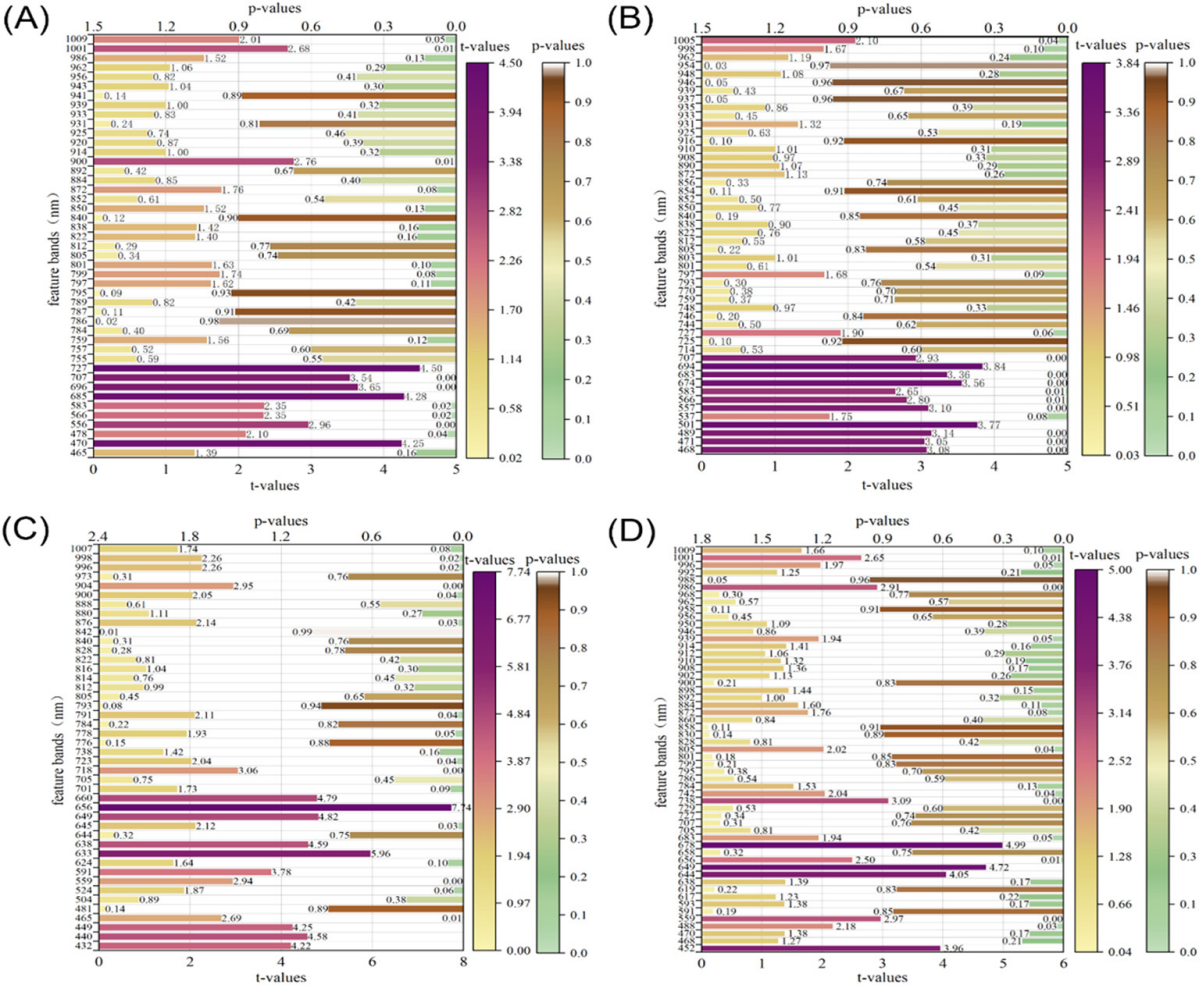
Significance tests for t-values and p-values of characteristic bands in the CARS-MLR model. **(A)** starch, **(B)** VC, **(C)** SSC, **(D)** TA.

The t-values and p-values are two key statistics used to assess the significance of regression coefficients. Usually, when the absolute value of t is more significant than two, or the absolute value of p is less than 0.05, it indicates that the effect of the variable on the target variable is substantial. As seen from **Figure 9**, there are 11, 12, 20, and 11 characteristic bands that significantly influence the starch, vitamin C, soluble solids, and titratable acid content in apple fruit, respectively. In addition, as shown in **Figure 9**, the characteristic bands with significant effects on starch and VC were all concentrated between 470 and 700 nm, and the absorption spectra of carotenoids and chlorophylls in apple fruit mainly caused the absorption peaks in this spectral interval. This finding further informed the potential relationship between starch content and physicochemical values of other nutrients.

## 4 Conclusions

In the wavelength range of 380-1018 nm, the internal nutrients, including VC, TA, SSC, and starch of apple fruit of different varieties, were detected non-destructively in this study using HSI. After comparing nine pretreatment methods, 2^nd^ Der was selected as the optimal pretreatment method in this study. To mitigate the effects of overlap and noise in the spectral curves on the changes in the feature information of the samples, three efficient data dimensionality reduction techniques, such as GA, SPA, and CARS, were used to screen out the wavelength combinations that could accurately characterize the changes in the feature information. This study constructed various prediction models such as SVR, PCR, PLSR, and MLR by using the full spectrum and the set of feature wavelengths as the input variables, respectively. The experimental results showed that the 2^nd^ Der-CARS-MLR model significantly outperformed the other models in predicting apples’ internal nutrient physicochemical indexes, which provided a generalized method for predicting different nutritional qualities. Meanwhile, the statistical analysis of t-values and p-values revealed that the characteristic bands affecting starch and VC content were in the same interval. The findings of this study suggest that the HSI of rotating samples is a non-destructive and efficient method suitable for detecting VC, TA, SSC, and starch in apple fruit, and the experimental results validate the feasibility of this method. However, this study analyzed only five apple varieties and one sample batch. For future scale-ups in complex commercial environments, more varieties, batches, and locations are planned to be utilized to build more robust predictive models.

## Data Availability

The original contributions presented in the study are included in the article/supplementary material. Further inquiries can be directed to the corresponding author.
